# Factors affecting attendance to cervical cancer screening among women in the Paracentral Region of El Salvador: a nested study within the CAPE HPV screening program

**DOI:** 10.1186/s12889-015-2360-7

**Published:** 2015-10-16

**Authors:** Karla M. Alfaro, Julia C. Gage, Alan J. Rosenbaum, Lauren R. Ditzian, Mauricio Maza, Isabel C. Scarinci, Esmeralda Miranda, Sofia Villalta, Juan C. Felix, Philip E. Castle, Miriam L. Cremer

**Affiliations:** Basic Health International, Colonia las Mercedes, Avenida los Espliegos #5, San Salvador, El Salvador; Division of Cancer Epidemiology and Genetic, National Cancer Institute, 9609 Medical Center Drive, Rockville, MD 20850 USA; Department of Obstetrics and Gynecology, University of Pittsburgh School of Medicine, 300 Halket Street, Pittsburgh, PA 15213 USA; Fulbright U.S. Student Program, U.S. Department of State, 1400 K Street NW, Suite 700, Washington, DC 20005 USA; Department of Obstetrics and Gynecology, The Ohio State University Wexner Medical Center, 395 W. 12th Ave., Columbus, OH 43210 USA; Division of Preventive Medicine, University of Alabama at Birmingham, Medical Towers 621, 1717 11th Avenue South, Birmingham, AL 35205 USA; Ministry of Health of El Salvador, Calle Arce, 827, San Salvador, El Salvador; Department of Pathology, University of Southern California, 2011 Zonal Ave., Los Angeles, CA 90033 USA; Global Cancer Initiative, 100 Radcliffe Drive, Chestertown, MD 21620 USA; OB/GYN & Women’s Health Institute, Cleveland Clinic 9500 Euclid Ave., Cleveland, OH 44195 USA

**Keywords:** HPV, Community health promoters, Cervical cancer, Adherence, Screening, Underserved populations, Education

## Abstract

**Background:**

Cervical cancer is the third most commonly occurring cancer among women and the fourth leading cause of cancer-related deaths in women worldwide, with more than 85 % of these cases occurring in developing countries. These global disparities reflect the differences in cervical cancer screening rates between high-income and medium- and low-income countries. At 19 %, El Salvador has the lowest reported screening coverage of all Latin American countries. The purpose of this study is to identify factors affecting public sector HPV DNA-based cervical cancer screening participation in El Salvador.

**Methods:**

This study was nested within a public sector screening program where health promoters used door-to-door outreach to recruit women aged 30–49 years to attend educational sessions about HPV screening. A subgroup of these participants was chosen randomly and questioned about demographic factors, healthcare utilization, previous cervical cancer screening, and HPV knowledge. Women then scheduled screening appointments at their public health clinics. Screening participants were adherent if they attended their scheduled appointment or rescheduled and were screened within 6 months. The association between non-adherence and demographic variables, medical history, history of cancer, sexual history, birth control methods, and screening barriers was assessed using Chi-square tests of significance and logistic regression.

**Results:**

All women (*n* = 409) enrolled in the study scheduled HPV screening appointments, and 88 % attended. Non-adherence was associated with a higher number of lifetime partners and being under-screened—defined as not having participated in cervical cancer screening within the previous 3 years (*p* = 0.03 and *p* = 0.04, respectively); 22.8 % of participants in this study were under-screened.

**Conclusions:**

Adherence to cervical cancer screening after educational sessions was higher than expected, in part due to interactions with the community-based health promoters as well as the educational session itself. More effective recruitment methods targeted toward under-screened women are required.

## Background

Cervical cancer is one of the leading causes of cancer-related mortality among women in low- and middle-income countries (LMICs) globally and in Latin America [1]. An estimated 529,828 cases and 275,125 deaths occur annually world-wide due to cervical cancer, with LMICs carrying more than 85 % of the burden of disease [2]. In Latin America and the Caribbean, the incidence and mortality rates are 21.2/100,000 and 8.7/100,000 respectively [3]. El Salvador has one of the highest rates of the region, with an incidence of 25/100,000 and a mortality rate of 11.8/100,000 [3].

El Salvador has a population of 2.41 million women, age 15 and older, who are at risk of developing cervical cancer in their lifetime, with current estimates of 823 new diagnoses and 388 deaths annually [4]. When compared to other Latin American countries, El Salvador has the lowest reported screening coverage. A National Family Health Survey (FESAL) 2008 report showed screening coverage to be 87.2 % overall, with 67.5 % of women screened every 2 years, and 45.0 % screened every year, but coverage levels decreased in the more rural areas of the country, with only 82.7 % of women having a history of screening, and 63.3 % of these women being screened within the last 2 years [4]. Among women with an abnormal screening result, follow-up is incomplete. According to a study conducted by Pan American Health Organization (PAHO) in 2002, 24 % (22/90) of women with abnormal Pap smear results did not receive a follow-up colposcopy [5].

To decrease this burden, numerous studies have tested interventions aimed at increasing cervical cancer screening with a particular focus on appointment attendance. Successful strategies have included scheduling appointments, mailing letters, making phone calls, conducting educational and counseling programs, and managing community-based outreach programs [6–8]. While most of these studies took place in developed countries, a few small-scale studies have been conducted in LMICs and demonstrated that cervical cancer education programs can increase knowledge, screening intention, and screening participation [9, 10].

Socio-demographic factors that have been shown to be associated with higher attendance rates in LMICs include relative higher wealth, seeking healthcare at health facilities when sick, and satisfaction with services at the health facility [11]. Structural and interpersonal barriers to screening include lack of knowledge of available services, financial constraints, family responsibilities, difficulty obtaining transportation, dissatisfaction with care, disapproval by a male partner, or discomfort with a male provider [11–16]. To date, studies of cervical cancer screening adherence in the context of a program that includes an educational session have not been performed among populations in rural Central America. Furthermore, the factors that influence women’s screening adherence rates in El Salvador are unidentified/unknown.

The purpose of this study is to identify the facilitators and barriers to HPV screening which could be instrumental when developing interventions for El Salvador as well as for other LMIC countries. The Cervical Cancer Prevention in El Salvador (CAPE) HPV screening program provided an opportunity to conduct this study within the initiative. CAPE was led by the Salvadoran Ministry of Health (MOH) in partnership with the non-profit organization, Basic Health International (BHI), to initiate HPV-based screening in the public sector using careHPV (QIAGEN Gaithersburg Inc., Gaithersburg, MD, USA) among approximately 30,000 women.

## Methods

This study was reviewed and approved by the Institutional Review Board of the University of Pittsburgh and the National Ethical Review Board of El Salvador.

The Factors Determining Screening Adherence Study (Adherence Study) was nested in the first phase of the CAPE program that introduced HPV screening to 2000 women at four rural health units in the Paracentral region of El Salvador (San Pedro Perulapan, San Rafael Cedros, Apastepeque, and San Sebastián). These units are responsible for providing primary preventive care. . The health promoters and MOH administration used the 2010 census to identify all women age 30–49 years potentially eligible for HPV testing in their catchment areas (*n* = 11,421). Health promoters are local employees of the MOH who reside in the communities in which they work and promote preventive health initiatives by providing education and counseling

**Fig. 1:**
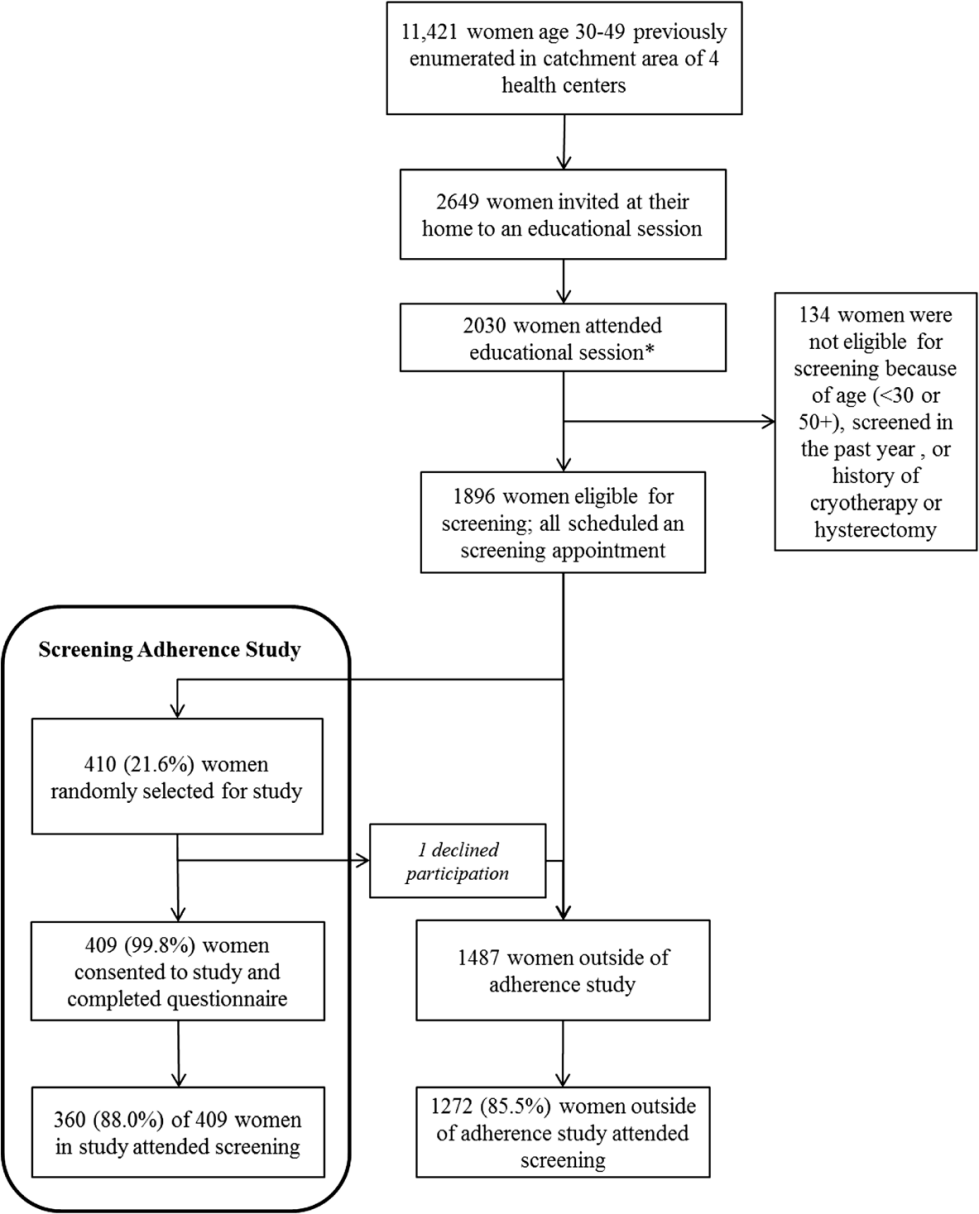
Recruitment and adherence to screening for Phase one of the CAPE project and the nested study of Factors Determining Screening Adherence, Paracentral Region, El Salvador

Figure 1 outlines the first phase of the CAPE program and the nested Adherence Study. The MOH-led CAPE program recruited women between October 2012 and March 2013 to receive HPV screening as part of an implementation program. Health promoters used health unit cytology registries to identify women not screened within the past 3 years and visited them in their home to promote screening. At the visits, many women (number not recorded in the CAPE program) reported that their screening history in the cytology registry was outdated because they had been screened in the past 3 years during a public health campaign or at some other occasion. Since this was an implementation rather than a research program, the Ministry of Health made the decision that screening would be based on age rather than screening history. This decision was made because preliminary data showed similar HPV positivity rates regardless of screening history. Therefore, all women age 30–49 years, not pregnant, able to provide informed consent, and without history of previous lesion or cryotherapy, loop electrosurgical excision procedure (LEEP), or hysterectomy were deemed eligible for HPV screening in the CAPE program (*n* = 2649).

Women were invited to attend an educational session in their community focusing on cervical cancer prevention and HPV testing. At this session, eligibility criteria were confirmed because some women attending had not been invited by a health promoter but instead self-referred outside of CAPE (number not reported in the CAPE program). A the educational sessions, a total of 1896 women were deemed eligible for HPV testing and were scheduled for screening appointments to take place at their local health clinics.

For this nested study, trained interviewers, hired specifically for this Adherence Study went to educational sessions conducted by each of the four health units (eight interviewers total) to randomly select one-third of the women attending until a target sample of 409 women enrolled. The Adherence Study stopped enrolling at a preset target of 409 screening participants. This number was chosen so that there was 80 % power to detect a difference of 15 % or greater in the appointment attendance between those who had a history of screening versus those who did not (i.e., had not been screened within the previous 3 years).

Upon arrival to the educational session, the first 18 women were given a card that was one of three colors, distributed in an alternating fashion; one color was chosen at random to select one-third of the women for invitation to the Adherence Study. Thus, a maximum of six interviews were performed at each educational session.

Prior to the start of the educational session, research assistants obtained informed consent from the selected women and conducted a brief interview. Women were asked about frequency of healthcare-related visits, reasons for visits, and barriers to healthcare utilization, and the distance, travel time, and method of transportation required to visit their healthcare providers. They were also asked about their previous cervical cancer screening history, knowledge of HPV and cervical cancer risk factors, and demographic information. Reasons for non-adherence to previous screening opportunities as well as reasons for non-adherence to clinic visits when ill were also elicited. The women then rejoined the rest of the group for the educational session, which was presented by either a clinician or a health promoter and covered topics such as HPV, cervical cancer, screening methods, HPV testing, self-sampling and provider-collected sampling, interpreting HPV results, and possible treatments.

As part of the CAPE program, all women who attended the educational talk were given the option of scheduling an appointment 15 days after the session. To verify whether women enrolled in the Adherence Study adhered to their screening appointment, research assistants reviewed the appointment book weekly to report women as having attended or missed their appointment. Women were considered adherent if they attend the scheduled appointment or if they missed the scheduled appointment but rescheduled and attended screening within the following 6 months.

Adherence to screening was calculated for both the women enrolled in the Adherence Study and all women recruited as part of the CAPE program. Within the Adherence Study, Chi-square tests of significance were used to assess the association between non-adherence and demographic variables (age, marital status, educational attainment, employment status, household size, and obstetrical parity); personal history of medical conditions (cancer, diabetes, heart disease, hypertension, and depression); family history of cancer; sexual history (age of sexual initiation, number of lifetime sexual partners, number of sexual partners in the past 3 months, and history of sexually transmitted infections); current birth control method; cervical cancer screening history (provider recommendation for cervical cancer screening within the past 3 years, time since last cervical cancer screening); and structural barriers to screening (distance and transportation time from their home to health clinic, type of transportation used to attend screenings, whether someone in their household owns a car). The significance level was set at 0.05 and all statistical analyses were conducted using STATA version 12.1 analytic software (StataCorp LP, College Station, TX). Logistic regression was used to explore the relationship between the independent variables listed above and adherence to screening. Adjusting variables with P values less than 0.10 in the bivariate analyses (Tables 1–3) were included in the unadjusted logistic regression models. Variables with *p*-values less than 0.10 in unadjusted models were entered into the reduced model using backwards elimination. Significance for variables in reduced models was set at *p* < 0.05.

**Table 1:** Socio-demographic characteristics and family history in Attendance Study, Paracentral Region, El Salvador, October 2012–March 2013

	Total		Attended screening		Did not attend		*p*-value^a^
	N	%	n	%	n	%	
Total	409	100.0	360	100.0	49	100.0	
Age (years)							
30–34	139	34.0	123	34.2	16	32.7	
35–39	122	29.8	105	29.2	17	34.7	
40–44	94	23.0	80	22.2	14	28.6	
45–49	51	13.2	52	14.4	2	4.1	.19
Highest education							
Elementary/none	216	52.8	190	52.8	26	53.1	
Middle school	153	37.4	133	36.9	20	40.8	
High school or higher	40	9.8	37	10.3	3	6.1	.69
Marital status							
Married	199	48.7	178	49.4	21	42.9	
Living together	121	29.6	102	28.3	19	38.8	
Single/widowed/separated	89	21.8	80	22.2	9	18.4	.32
Number of children							
0–2	113	27.6	101	28.1	12	24.5	
3–4	178	43.5	155	43.1	23	46.9	
> 5	118	28.9	104	28.9	14	28.6	.84
Size of household							
1–3	20	4.9	18	5.0	2	4.1	
4–5	137	33.5	117	32.5	20	40.8	
> 6	252	61.6	225	62.5	27	55.1	.51
Work outside home	95	23.2	82	22.8	13	26.5	.56
Provider has told you that you have:							
Cancer	4	1.0	4	1.1	0	0.0	1.0
Diabetes	14	3.4	12	3.3	2	4.1	.68
Heart disease	15	3.7	14	3.9	1	2.0	1.0
High blood pressure	50	12.2	42	11.7	8	16.3	.38
Depression	46	11.3	40	11.1	6	12.2	.81
Family member has had cancer	51	12.5	43	11.9	8	16.3	.38

^a^Chi-square or Fisher’s exact test

**Table 2:** Sexual history and screening experience of participants for screening attendance

	Total		Attended screening		Did not attend		*p*-value^a^
	n	%	n	%	n	%	
Total	409	100.0	360	100.0	49	100.0	
Age of first intercourse (years)							
< 16	83	20.5	75	21.1	8	16.3	
16–19	210	51.9	189	53.1	21	42.9	
> 20	112	27.7	92	25.8	20	40.8	.09
Number of lifetime sexual partners							
1	189	47.0	167	47.2	22	45.8	
2–3	178	44.3	161	45.5	17	35.4	
> 4	35	8.7	26	7.3	9	18.8	.03
Number of partners in past 3 months							
Zero	57	14.1	54	15.2	3	6.3	
One	343	85.1	299	84.2	44	91.7	
Two or more	3	0.8	2	0.6	1	2.0	.10
Current birth control method							
1 month injection	13	3.2	12	3.3	1	2.0	
2 month injection	33	8.1	29	8.1	4	8.2	
3 month injection	57	14.0	45	12.5	12	24.5	
Tubal ligation	88	21.6	80	22.3	8	16.3	
Other	20	4.9	16	4.5	4	8.2	
None	197	48.3	177	49.3	20	40.8	.21
Ever had an STI	71	17.4	61	17.0	10	20.4	.59
Provider recommended screening in past 3 years	265	64.8	237	65.8	28	57.1	.23
Last screen for cervical cancer							
</=3 years ago	312	77.2	281	78.9	31	64.6	
> 3 years ago	81	20.1	67	18.8	14	29.2	
Never	11	2.7	8	2.3	3	6.3	.04

^a^Chi-square or Fisher’s exact test

**Table 3:** Logistical barriers for participation and screening adherence

	Total		Attended screening		Did not attend		*p*-value^a^
	n	%	n	%	n	%	
Total	409	100.0	360	100.0	49	100.0	
Distance to clinic from home							
< 10 km	330	80.7	295	81.9	35	71.4	
10–20 km	39	9.5	33	9.2	6	12.2	
> 20 km	40	9.8	32	8.9	8	16.3	.18
Main transport method to clinic^b^							
Walking	68	16.6	58	16.1	10	20.4	
Personal vehicle	6	1.5	6	1.7	0	0.0	
Friend’s vehicle	30	7.3	25	6.9	5	10.2	
Public transport	305	74.6	271	75.5	34	69.4	.56
Someone in home owns a car	58	14.2	51	14.2	7	14.3	.98
Time to get to clinic from home							
< 5 min	38	9.3	36	10.0	2	4.1	
> 5–30 min	218	53.3	195	54.2	23	46.9	
> 30–60 min	95	23.2	79	21.9	16	32.7	
> 60 min	58	14.2	50	13.9	8	16.3	.23

^a^Chi-square or Fisher’s exact test

^b^1 woman reported friend’s vehicle

## Results

Among 1896 women who attended the education session for the CAPE program and were eligible for screening, 410 (21.6 %) were randomly selected to participate in the Adherence Study and all but one (99.8 %) provided consent (Fig. 1). Adherence to screening was similar between women participating in the Screening Adherence study (88.0 % of 409) compared to women not participating (85.5 % of 1487; *p* = 0.23) (Fig. 1).

Among the 409 women who participated in the Adherence Study, demographic characteristics, personal history of cancer, and screening attendance are presented in Table 1. The average age of participants was 37.6 years, and the majority were either married or living with their partner. Most had more than three children, lived in a household with at least six other individuals, had not completed a middle school education, and did not work outside of the home.

Sexual history, screening experience, and the associations with attendance are presented in Table 2. More than half of participants reported first sexual activities occurred between 16 and 19 years of age, and 85 % of participants reported sexual activity with one partner within the last 3 months. Approximately 50 % of participants reported one lifetime partner, and most of the remaining women reporting 2–3 lifetime partners (44.3 %; range of 1–50 partners). Only 1.5 % of patients reported any condom use, and 17.4 % reported having had a previous sexually transmitted infection. A majority of participants stated that a provider had recommended a cervical cancer screening within the past 3 years (64.8 %), and 77.2 % of women reported having participated in screening within the past 3 years. A higher number of lifetime sexual partners and a longer period of time since the last cervical cancer screening were the only variables significantly associated with non-attendance to the screening appointment (*p* = 0.03 and *p* = 0.04, respectively).

Adherence and structural barriers to cervical cancer screening are presented in Table 3. At the interview, 100.0 % of patients reported willingness to attend screening; 88.0 % attended their screening appointment. The vast majority lived less than 10 km from a health clinic and required public transportation for travel. Although most women required less than a half hour of travel time to reach the clinic, 14.2 % of patients reported requiring more than 1 h to travel to the clinic.

These findings were consistent when adjusting for age. Multivariate analysis of variables potentially associated with adherence (including age) showed that after adjustment, having four or more sexual partners, a time period of greater than 3 years from previous screening, or never having had screening continued to be statistically associated with non-adherence (Table 4).

**Table 4:** Odds ratios (OR) and 95 % confidence intervals (CI) of attending screening for factors associated with screening attendance

	Univariate OR (95 % CI) of attending screening		Multivariate OR (95 % CI) of attending screening	
	Odds ratio (OR)	95 % Confidence Interval (CI)	Odds ratio (OR)	95 % Confidence Interval (CI)
Age of first intercourse (years)				
< 16	1.00	−	−	−
16–19	1.05	0.45–2.48	−	−
> 20	2.18	0.90–5.27	−	−
Number of lifetime sexual partners				
1	1.00	−	1.00	−
2–3	0.80	0.41–1.56	0.74	0.37–1.46
> 4	2.60	1.08–6.28	2.69	1.10–6.56
Last screen for cervical cancer				
</=3 years ago	1.00	−	1.00	−
> 3 years ago	2.00	1.00–4.01	2.06	1.02–6.56
Never	3.32	0.83–13.2	4.78	1.14–19.95

Under-screened women were asked specifically about why they had not been screened recently, and women reported that cervical cancer screening in particular was painful (61.4 %), uncomfortable or embarrassing (64.3 %), and women did not like having to see male providers (71.4 %). Many women were unsure of the purpose of a Pap test (61.4 %), did not recall any suggestions around screening from their providers (65.7 %), or believed that cervical cancer screening was unnecessary (65.7 %).

## Discussion

This study examined factors associated with adherence to a scheduled appointment for cervical cancer screening using HPV DNA testing. The most important finding of this study was that the attendance rate was excellent—88.0 % of women in this study attended their scheduled appointment. Programmatic influences that may have contributed to the high appointment adherence include interaction with local health promoters, the educational seminar, a novelty effect in that the test was new to the region, and the fact that the program was advertised as potentially allowing women to not require further screening for at least 5 years. Some of these variables have been elicited in past studies that have found educational programs to increase screening participation [6–9].

The design of the CAPE program involved health promoters meeting women both at their homes and at the educational session. This outreach method is standard for the Salvadoran MOH and has been increasing globally, with the governments of Nepal, Ethiopia, Ghana, and Iran aiming to use door-to-door outreach on a national level. In our study, we found an unanticipated factor was the enthusiasm of the health promoters: without instruction from researchers, many health promoters displayed initiative in reminding women of upcoming appointments, contacted patients to reschedule if the previous appointment was missed, or helped provide transportation for women to attend the educational sessions and screening appointments. As a result, logistical barriers were not associated with a significant decrease in adherence to screening. The level of interaction varied among health promoters, but having local representatives communicate with women repeatedly throughout the scheduling and screening process clearly contributed to the high rate of attendance. This finding is in accord with findings from previous studies regarding communication with patients prior to scheduled appointments [6–8].

The use of health promoters in the implementation of screening programs is a sustainable strategy over time, especially in the context of El Salvador. Past studies have shown the benefit of using educational programs to increase screening rates, and this study further reinforces the role of health promoters in achieving this public health goal [6–9]. Health promoters are women employed by the Ministry of Health responsible for educating and working with a given number of people within a service area. The focus of their work is on the prevention of cervical cancer, and their efforts and training are part of a bigger plan to scale up CAPE at a national level. Cervical cancer prevention and screening programs have shown evidence of greater patient follow-through when the target community is directly involved in the implementation of services [17]. These contributions likely helped to increase our adherence rate and provide a guide for programmatic improvements in the future.

It should be noted that the two factors that were associated with non-adherence to screening were higher number of lifetime sexual partners and longer time since last cervical cancer screening, indicating that those with greatest need for screening were least likely to attend. Prior literature finds that women with high-risk behavior including cigarette smoking, unprotected sexual intercourse, acute alcohol consumption, and women with multiple sexual partners are less likely to seek preventive medical interventions [18, 19]. Certain high-risk behaviors such as smoking and alcohol consumption were not studied in detail here. Smoking, alcohol abuse, and engaging in high-risk sexual behavior, such as having multiple sexual partners, are not common practices amongst women in El Salvador and therefore do not contribute significantly to cervical carcinogenesis in this population [4]. A previous study conducted in the same region of El Salvador found that very few women in the area had ever smoked [20].

Interestingly, our study did not show an association between screening adherence and use of contraception, age, and education as other studies have. The fact that the majority of women in this study had been recently screened might explain the lack of observed association.

An important limitation of this study was that the population included was likely not representative of all women from a rural Salvadoran community. By design, women not attending the educational session could not participate in the study. These constituted perhaps 23 % of women in the rural community. It is likely that women failing to attend an educational session were least likely to attend screening. This selection bias is evidenced in the high proportion of women enrolled in the adherence study who received screening in the past 3 years (77 %). This is higher than expected from a country where 1-year cervical cancer coverage estimates range from 17 to 42 % [21, 22]. This limitation is pervasive in cervical cancer screening studies which are highly populated by healthcare seekers. In this instance, the behavior may have uncovered a converse behavior with health promoters seeking women who will comply. Regardless, the data identifies a few important areas (e.g., educational sessions and home-based recruitment by health promotors) improved adherence that should be addressed in the future. In addition, El Salvador is a fairly homogenous country and the CAPE program was designed to capture most of the Paracentral region which we expect to generalize to the whole country.

## Conclusions

In conclusion, the educational sessions by individuals who live in these communities and the use of health promoters may be effective in recruiting and scheduling HPV screening appointments. Overall, adherence to screening was high, yet women with a history of non-screening as well as those with more lifetime sexual partners were less likely to attend. Further studies are needed to identify effective recruitment methods targeted at this at-risk population.
